# Transglutaminase-2 mediates acquisition of neratinib resistance in metastatic breast cancer

**DOI:** 10.1186/s43556-022-00079-y

**Published:** 2022-06-22

**Authors:** Aparna Shinde, Eylem Kulkoyluoglu Cotul, Hao Chen, Andrew Smith, Sarah Libring, Luis Solorio, Michael K. Wendt

**Affiliations:** 1 Department of Medicinal Chemistry and Molecular Pharmacology, Purdue University, West Lafayette, IN 47907 USA; 2 Department of Biomedical Engineering, Purdue University, West Lafayette, IN 47907 USA; 3 Purdue Center for Cancer Research, Purdue University, West Lafayette, IN 47907 USA

**Keywords:** Breast cancer, Drug resistance, Metastasis, HER2, IL-6

## Abstract

Acquisition of resistance to targeted therapies remains a major clinical obstacle for the HER2^+^ subtype of breast cancer. Using an isogeneic progression series of HER2^+^ breast cancer metastasis we demonstrate that metastatic cells have an increased capacity to acquire resistance to the covalent, pan-ErbB inhibitor, neratinib. RNA sequencing analyses comparing parental and metastatic cells identified upregulation of transglutaminase 2 (TG2). Genetic depletion and overexpression approaches established that TG2 is both necessary and sufficient for acquisition of neratinib resistance. Mechanistically, we describe a pathway in which TG2-mediates activation of NF-κB signaling leading to upregulation of IL-6 in metastatic cells. This autocrine expression of IL-6 functions to maintain enhanced levels of TG2 via JAK:STAT3 signaling. This drug persistence feedback loop can be interrupted through the use of the JAK1/2 inhibitor ruxolitinib. In vivo application of ruxolitinib had no effect on tumor growth under non-treated conditions, but effectively prevented acquisition of resistance, leading to tumor regression upon coadministration with neratinib. Overall, our studies reveal a mechanism in metastatic breast cancer cells that predisposes them to acquisition of resistance to ErbB-targeted therapeutics. Clinically, immediate application of ruxolitinib could prevent acquisition of resistance and improve patient responses to HER2-targeted therapies.

**Supplementary Information:**

The online version contains supplementary material available at 10.1186/s43556-022-00079-y.

## Introduction

Human epidermal growth factor receptor-2 (HER2) is overexpressed in approximately 20% of breast cancers. The ability to clinically identify this driver event has led the development and effective application of a growing variety of HER2-targeted agents including antibodies, antibody drug conjugates and kinase inhibitors [1]. Despite the clinical success of HER2-targeting agents, the presence of drug persistent minimal residual disease and recurrence of fully drug resistant tumors frequently occurs in the metastatic setting [2–4].

Neratinib is a covalent kinase inhibitor approved for use in HER2^+^ breast cancer in 2017 [5]. Neratinib is a highly potent compound that has significant inhibitory activity against EGFR, HER2 and ErbB4 [6]. We have demonstrated that prolonged treatments with the first generation HER2/EGFR competitive kinase inhibitor, lapatinib, can result in spontaneous acquisition of resistance. In contrast, treatment with neratinib and the related covalent ErbB inhibitor, afatinib, fails to result in spontaneous resistance [7]. The reasons for the sustained efficiency of neratinib are not completely clear, but neratinib does target an increased number of kinases outside of the ErbB family as compared to lapatinib. Additionally, unlike lapatinib, neratinib induces the degradation of HER2 [8, 9].

Tissue transglutaminase 2 (TG2) plays several roles in tumor biology where it is secreted and acts as a crosslinking enzyme capable of stabilizing the fibrillar nature of the extracellular matrix [10]. We and others have also demonstrated that TG2 is present on tumor cell-derived extracellular vesicles and participates in the creation of pre-metastatic niches [11, 12]. In addition to the extracellular activities, TG2 is also active inside the cell where it crosslinks Iκbα making it unavailable for cytoplasmic sequestration of Nuclear factor-kappa-B (NF-κB) [13, 14]. Upregulation of proinflammatory NF-κB signaling plays important roles in sensing and contributing to changes in the tumor microenvironment (TME). Activation of NF-κB signaling has also been linked to lapatinib resistance and resistance to the anti-HER2 therapeutic antibody trastuzumab [15, 16]. Despite these findings the mediators of NF-κB activation and the function of this pathway in response to the next generation kinase inhibitors remained to be determined.

A critical downstream output of NF-κB activation is the upregulation of interleukin-6 (IL-6). This cytokine can be produced by a number of stromal cell types and acts on tumor cells in a paracrine fashion. In addition, tumor cells can also produce IL-6 to establish an autocrine signaling axis. IL-6 and downstream activation of JAK2/STAT3 signaling play critical roles in tumor progression, establishment of a stem-like phenotype, and drug resistance [16]. Importantly, this pathway can be readily blocked via application of clinically approved IL-6 blocking antibodies and JAK inhibitors, such as ruxolitinib [17].

In the current study, we establish that in metastatic HER2^+^ breast cancer cells upregulation of TG2 is an initiating component of the NF-κB:STAT3 signaling loop allowing cells to persist in the presence of neratinib. We utilize a number of genetic and pharmacological approaches to demonstrate the importance of this feedback signaling loop in facilitating acquisition of neratinib resistance. Overall, our studies support the notion that combining ruxolitinib with HER2-targeted agents could inhibit the development of drug resistance in the metastatic setting and prolong patient response to ErbB-targeted agents.

## Results

### Transglutaminase 2 facilitates acquired resistance to neratinib

**Fig. 1:**
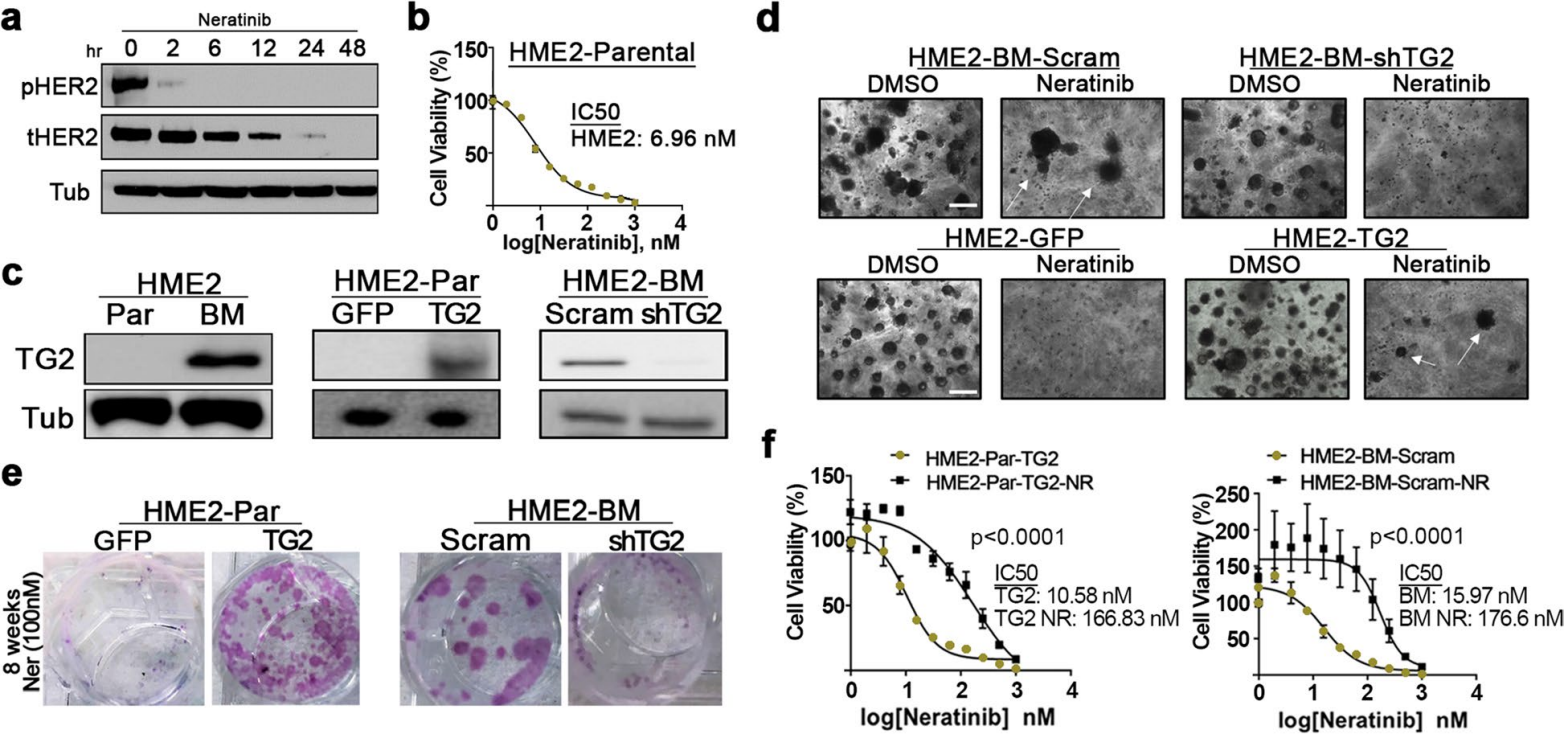
Transglutaminase 2 facilitates neratinib resistance. **a** HME2-partenal cells were treated with neratinib (1 µM) for the indicated amounts of time and phosphorylated (p) and total (t) HER2 were evaluated by immunoblot. β-tubulin was used as a loading control. **b** HME2-parental cells were treated (96 h) with neratinib at the indicated concentrations and cell viability was assayed. Data are the mean ± SE of three independent experiments resulting in the indicated IC50 value. **c** Immunoblot analyses for TG2 in HME2 parental (Par) and bone metastases (BM). HME2-GFP, HME2-TG2, HME2-BM-scram and HME2-BM-shTG2 were also assessed for TG2 expression. β-tubulin was used as a loading control. Data are representative of at least three independent experiments. **d** TG2 manipulated cells were seeded under single-cell 3D culture conditions in the presence of DMSO or neratinib (100 nM). Images of each condition at day 27 are shown. **e** TG2 manipulated cells were treated in 2D culture every three days with neratinib (100 nM) for a period of 8 weeks. Crystal violet was used to stain representative wells with at the indicated time points to visualize drug resistant cells. **f** The neratinib resistant (NR) cells were cultured for an additional 4 weeks without neratinib. The drug resistant cells and passage matched HME2-BM-Scram and HME2-Par-TG2 cells were treated with the indicated concentrations of neratinib for 96 h and cell viability was assayed. Data are the mean of three independent experiments ± SE, resulting in the indicated IC50 and *P* values

Previous studies from our lab and others demonstrate that directed overexpression of HER2 is sufficient to transform the immortalized human mammary epithelial (HMLE) cell line [7, 18]. These HER2 transformed cells (HME2) are sensitive to treatment with ErbB inhibitors including neratinib, which potently inhibits HER2 phosphorylation and leads to degradation of the receptor (Fig. 1a and 1b) [8]. In our previous studies, we utilized RNA sequencing analyses to characterize gene expression differences between the parental HME2 population and their isogenic derivatives that were isolated and subcultured from bone metastases (HME2-BM) [11, 19]. These analyses indicate that TG2 is part of a 1150 gene signature that is significantly regulated in the HME2-BM cells as compare the HME2 parental population. In addition to these mRNA analyses this increase in TG2 expression can also be readily observed at the protein level (Fig. 1c) [11]. To characterize the functional role of TG2 in response to neratinib we depleted its expression in the BM cells and overexpressed it in the HME2 parental population (Fig. 1c) [11]. In contrast to the HME2 parental population, the HME2-BM cells were capable of spontaneously developing resistance to neratinib upon prolonged drug treatment (Fig. 1d). Using a 3D culture approach we could readily observe the formation of drug resistant HME2-BM cell clusters upon 4 weeks of treatment with neratinib (Fig. 1d). Importantly, acquisition of neratinib resistance was abolished upon depletion of TG2 (Fig. 1d). Furthermore, directed overexpression of TG2 in the HME2 parental cells resulted in the formation of drug resistant cell clusters (Fig. 1d). Similar to what was observed using 3D culture, TG2 was both necessary and sufficient for acquisition of neratinib resistance under 2D culture conditions (Fig. 1e). Expansion of these neratinib resistant populations allowed us to quantify greater than tenfold increases in IC50 values compared to drug sensitive counterparts (Fig. 1f).

**Fig. 2:**
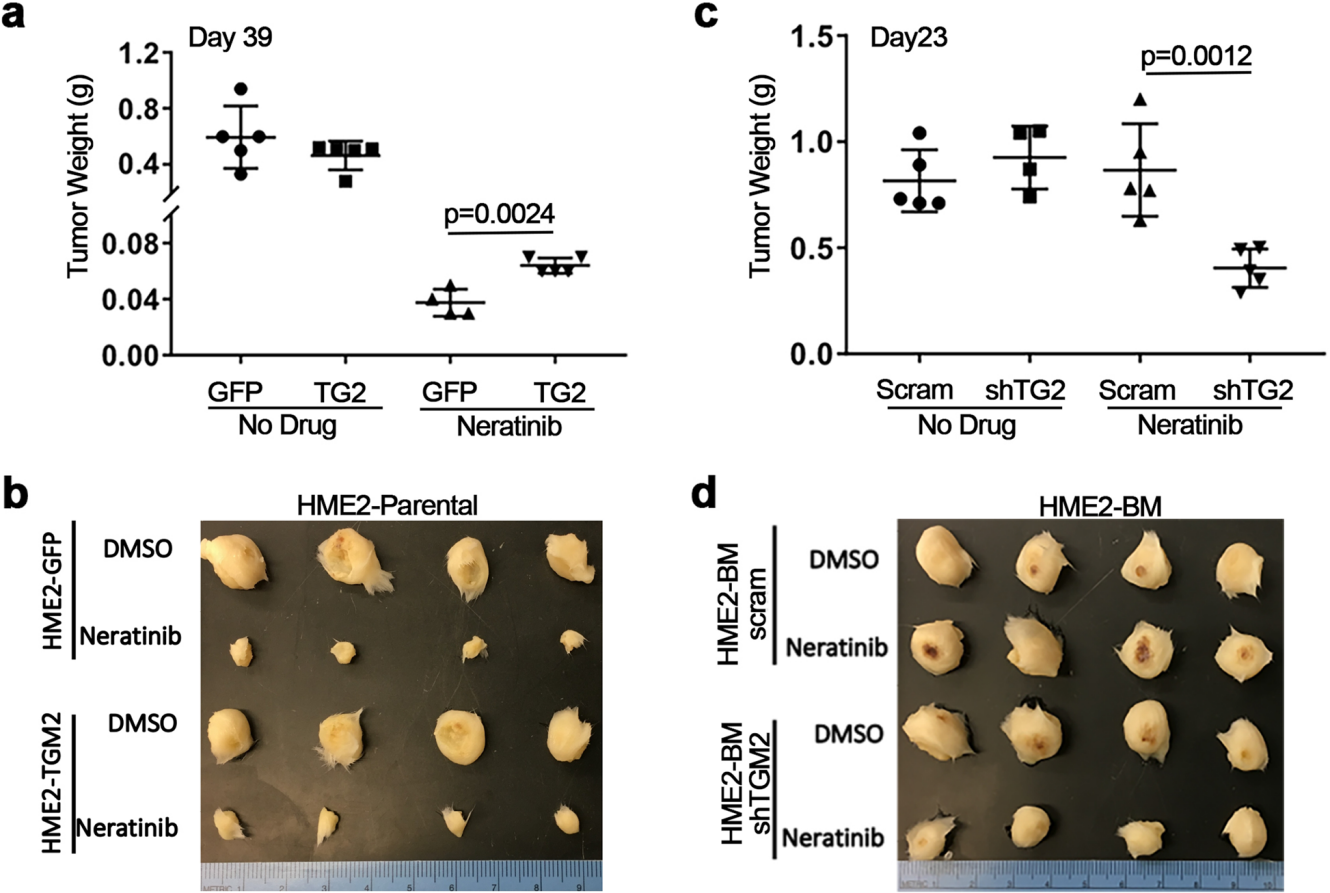
Transglutaminase 2 facilitates neratinib resistance in vivo. **a,c** HME2 parental cells expressing GFP or TG2, and HME2-BM cells expressing a control (scram) or TG2-targeted shRNA (shTG2) were injected into the mammary fat pad. Tumor bearing mice were treated with neratinib as described in the material and methods tumors were removed and weighed upon necropsy at the indicated time points. Data are the mean of 5 mice per group ± SE, resulting in the indicated *p*-values. **b,d** Upon necropsy, tumors were fixed and imaged

To validate the in vivo function of TG2 in drug resistance we engrafted TG2-manipulated HME2 and HME2-BM cells onto the mammary fat pad of NSG mice. Upon tumor establishment, mice were treated with neratinib via oral gavage, and the experiment was terminated when the non-treated tumors reached an average of 1000 mm^3^. The HME2 parental tumors required 39 days to reach 1000 mm^3^ (Fig. 2a-b). In contrast, the HME2-BM tumors only required 23 days (Fig. 2c-d). This differential primary tumor growth rate was not affected by TG2 expression as neither depletion of TG2 in the HME2-BM cells nor overexpression of TG2 in the HME2 parental cells affected untreated tumor weights (Fig. 2a and b). Treatment with neratinib abolished HME2-parental tumor formation at a dose that did not cause adverse effects to the animals (Fig. 2a-b). Overexpression of TG2 diminished the effectiveness of neratinib at this same dose (Fig. 2a-b). In contrast to the HME2-parental tumors, treatment of the HME2-BM tumors with neratinib did not significantly affect tumor growth, but depletion of TG2 allowed for a significant inhibition of tumor growth upon neratinib treatment (Fig. 2c-d). Taken together these data clearly indicate that TG2 facilitates acquisition of neratinib resistance under 2D, 3D, and in vivo conditions.

### Transglutaminase 2 causes increased expression of IL-6

**Fig. 3:**
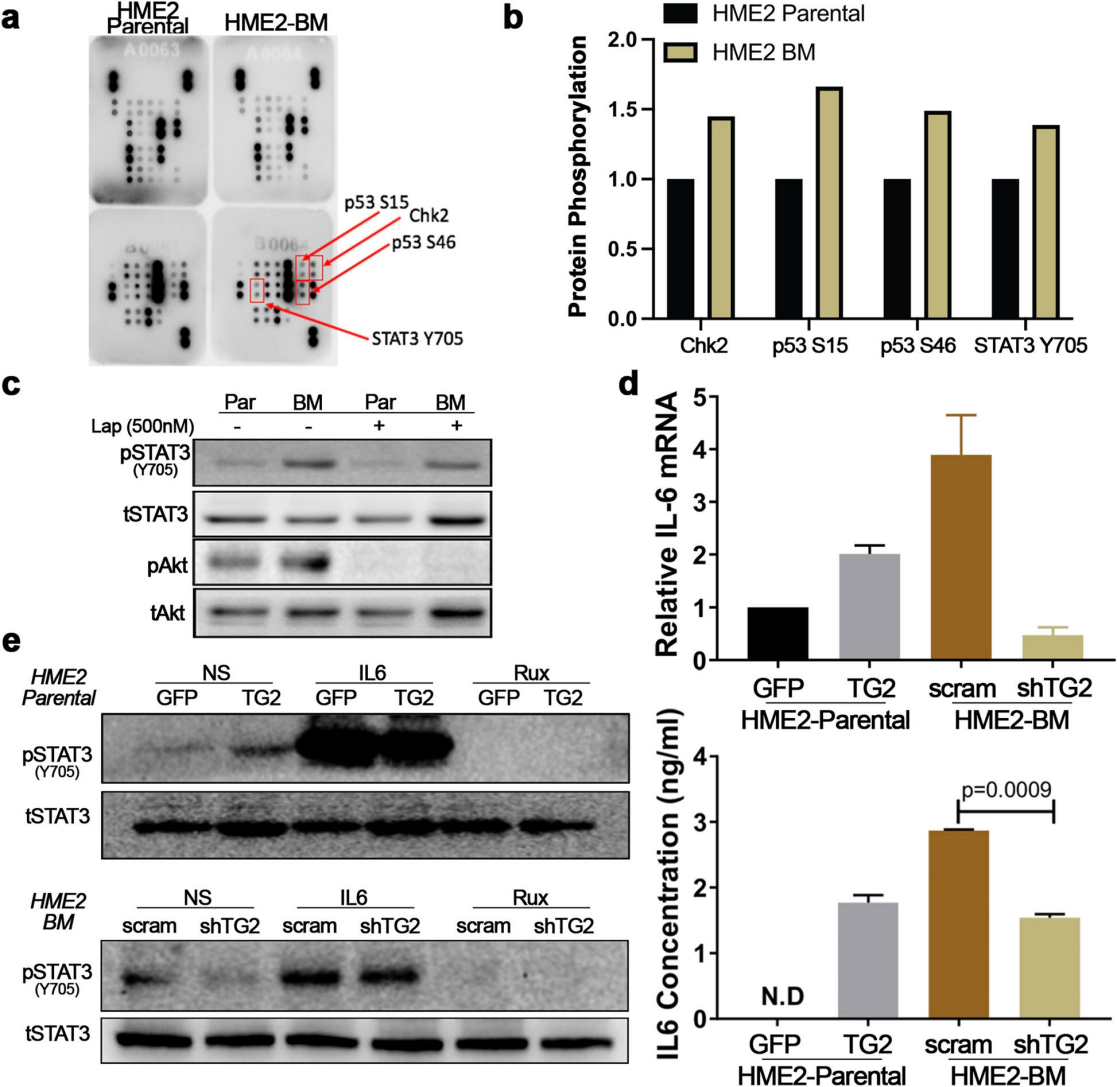
Transglutaminase 2 causes increased expression of IL-6. **a** Phosphorylation array comparing lysates derived from HME2-parental and HME2-BM cells. Proteins whose phosphorylation was potentially different between the two cell lines are indicated. **b** Spot intensity for the indicated sites of phosphorylation. Intensity values were normalized to the HME2-parental cells. **c** HME2-parental and HME2-BM cells were treated with Lapatinib (500 nM) for 2 h and analyzed for phosphorylation of STAT3 and AKT. Analysis of total levels of STAT3 and AKT served as loading controls. Immunoblots are representative of at least three independent experiments. **d** Transcript (upper graph) and protein (lower graph) levels for IL-6 in HME-parental and HME2-BM manipulated for TG2 expression were quantified using qRT-PCR and AlphaLISA. Transcript levels are expressed relative to GFP expressing HME2-parental cells. All data are the mean ±SE of three independent experiments resulting in the indicated *p*-values. **e** HME2-parental and HME2-BM cells manipulated for TG2 expression were stimulated with IL-6 (20 ng/ml) for 30 min or treated with ruxolitinib (1 µM) for 6 h and analyzed for phosphorylation of STAT3. Analysis of total levels of STAT3 served as loading controls. Immunoblots are representative of at least three independent experiments

We next sought to identify signaling pathways, other than HER2, that are active in the HME2-BM cells as compared to the HME2-parental. To this end we conducted a kinase array on lysates derived from the two isogenic cell lines. This approach suggested increased phosphorylation of p53, Chk2, and STAT3 (Fig. 3a-b). Consistent with this increase in STAT3 phosphorylation, examination of our gene expression analyses comparing the HME2-parental and BM cells identified upregulation of interleukin 6 (IL-6) in the HME2-BM cells (GSE115255). The enhanced phosphorylation of STAT3 in the HME2-BM cells was verified by immunoblot, and this event was not affected by treatment with the HER2/EGFR inhibitor lapatinib, at concentrations that abolished downstream phosphorylation of Akt in both the HME2-parental and HME2-BM cells (Fig. 3c). Increased expression and secretion of IL-6 in the HME2-BM cells were validated using RT-PCR and AlphaLISA assays (Fig. 3d). These analyses also indicated that directed overexpression of TG2 increased IL-6 in the HME2-parental cells and depletion of TG2 decreased IL-6 expression in the HME2-BM cells (Fig. 3b). Finally, we verified the functionality of this signaling axis in the HME2 cells through treatment with exogenous IL-6 and the JAK1/2 inhibitor ruxolitinib [17]. Clearly, both cells lines are capable of responding to IL-6 and the BM cells signal through JAK2 to phosphorylate STAT3 (Fig. 3e). Overall, these data indicate that increased expression of TG2 in metastatic cells can induce IL-6, leading to constitutive JAK:STAT3 signaling.

### Transglutaminase 2-mediated activation of NF-κB is required for neratinib resistance

**Fig. 4:**
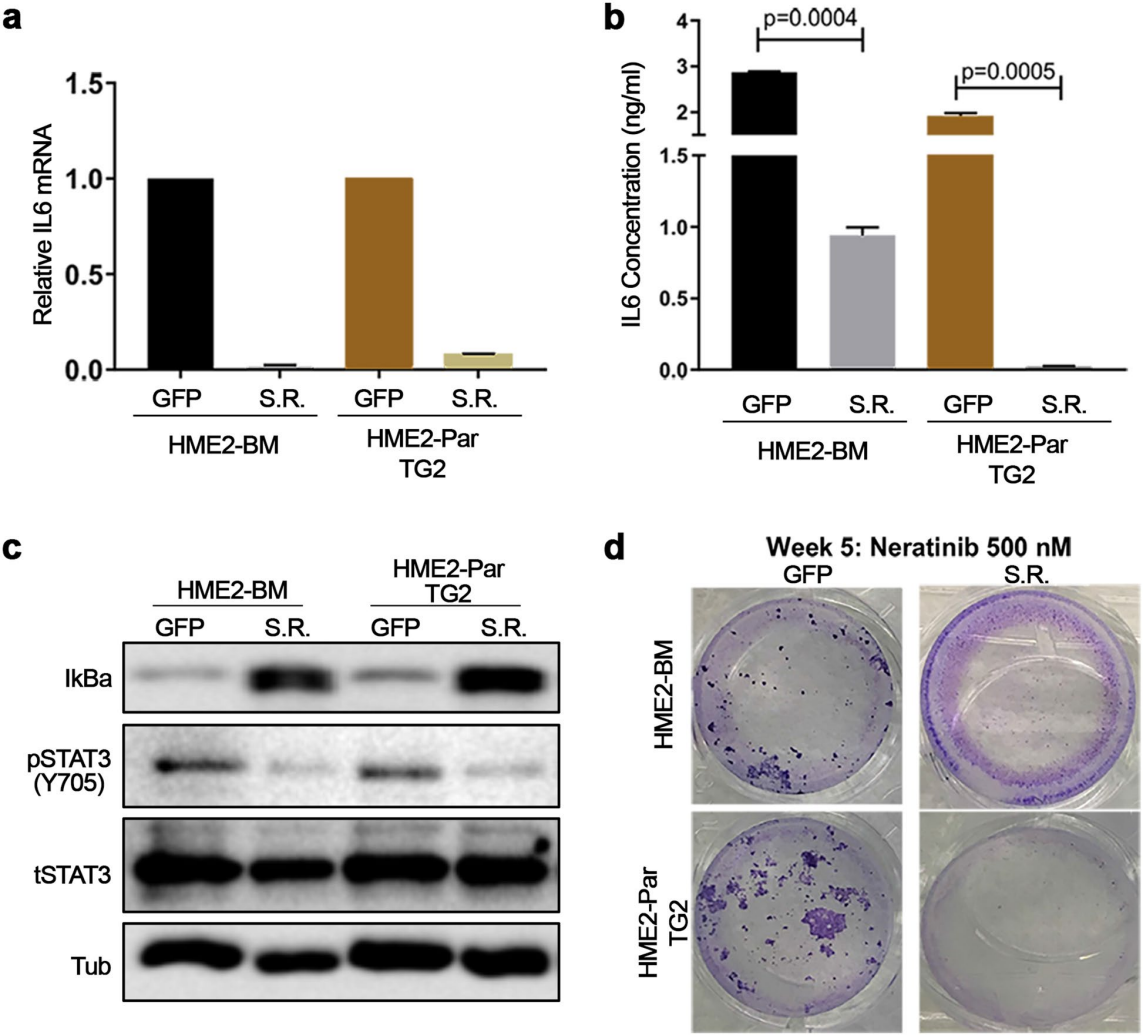
Transglutaminase 2-mediated activation of NF-κB is required for acquisition of neratinib resistance. **a,b** HME2-BM cells and TG2 overexpressing HME2-Parental (Par) cells were further constructed to express the NF-κB super repressor (S.R.) or GFP as a control and IL-6 was quantified using qRT-PCR (**a**) and AlphaLISA (**b**). **c** The constructed cell lines described in panel a in were serum starved overnight and analyzed for Iκbα (IkBa) expression and phosphorylation of STAT3. Analysis of total levels of STAT3 and β-tubulin served as loading controls. Immunoblots are representative of at least three independent experiments. **d** The constructed cell lines described in panel a were treated with neratinib every three days. Crystal violet was used to visualize viable cells in representative wells

TG2 has previously been linked to activation of NF-κb and this pathway is a well-established mediator of IL-6 expression [20, 21]. Consistent with these studies, we observed a significant decrease in NF-κb activity when TG2 was depleted (Supplementary Fig. 1). To elucidate if NF-κB activation is the mechanism by which TG2 causes increased expression of IL-6 we overexpressed the super repressor (S.R.), a non-degradable form of IκBα that prevents NF-κB translocation into the nucleus. Indeed, expression of the S.R. reduced expression of IL-6 in both the HME2-BM cells and the TG2 overexpressing HME2 parental cells (Fig. 4a-b). Accordingly, expression of the S.R. reduced STAT3 phosphorylation in both the HME2-BM cells and the TG2-overexpressing HME2-parental cells (Fig. 4c). Importantly, expression of the S.R. prevented spontaneous acquisition of resistance to neratinib upon prolonged culture in the presence of drug (Fig. 4c-d). Taken together, these data suggest a mechanism by which TG2-mediated activation of NF-κB increases autocrine IL-6 expression leading constitutive activation of STAT3, allowing for acquisition of resistance to HER2-targeted therapies.

### STAT3-driven expression of transglutaminase-2 drives drug resistance

**Fig. 5:**
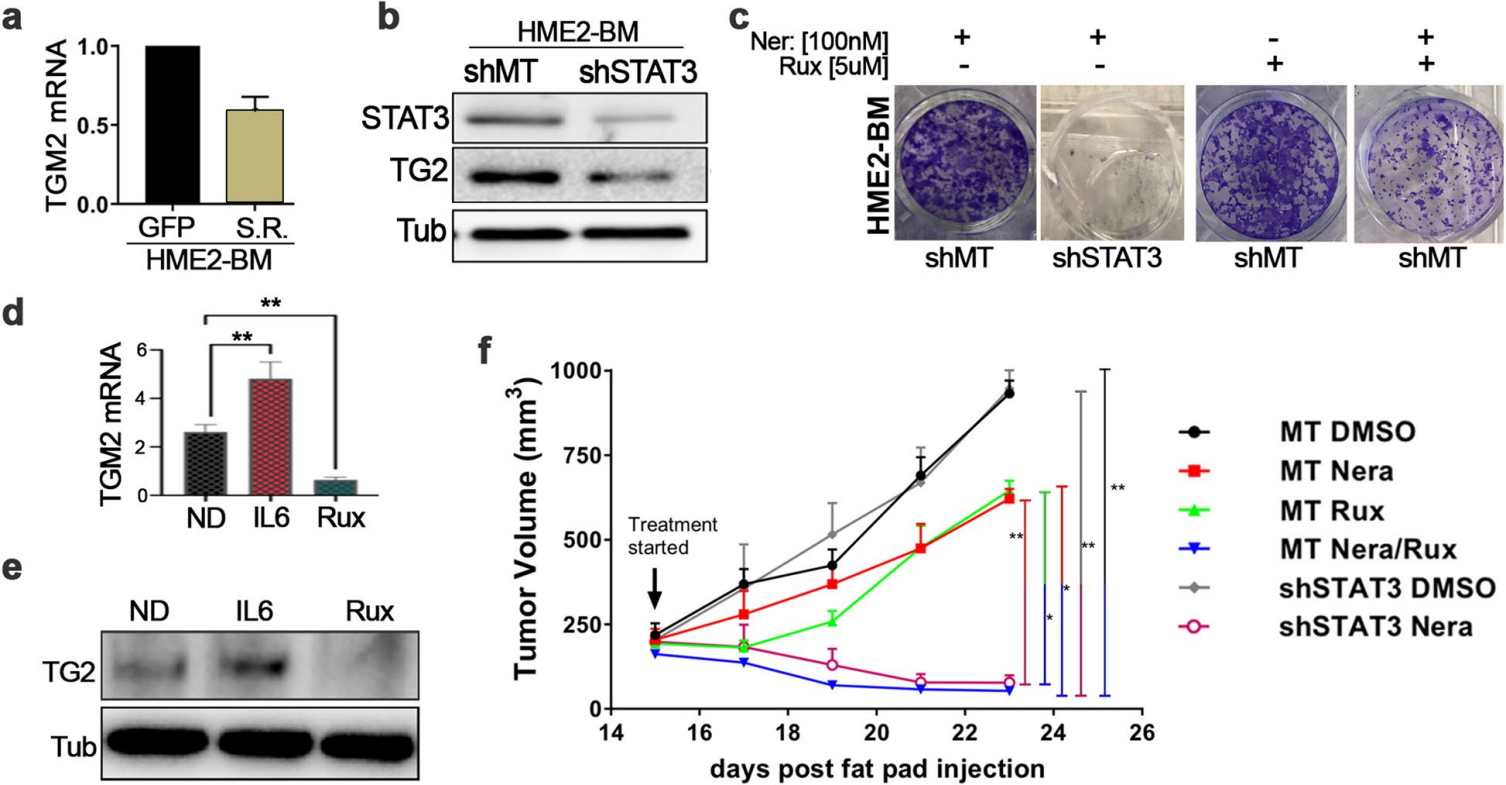
STAT3-driven expression of transglutaminase-2 faciliates drug resistance. **a** Transcript levels for TG2 in HME2-BM cells expressing the NF-κB super repressor (S.R.) or GFP as a control were quantified using qRT-PCR. Data are relative to empty (MT) vector control (HME2-BM-MT) cells. **b** HME2-BM cells expressing a empty shRNA vectors (shMT) one those targeting STAT3 (shSTAT3) were analyzed for TG2 and STAT3. Analysis of β-tubulin served as a loading control. Immunoblots are representative of at least three independent experiments. **c** HME2-BM cells described in panel b were treated with neratinib, ruxolitinib, or both drugs every three days for a period of 5 weeks. Crystal violet staining was used at the indicated time points to visualize resistant cells. **d** HME2-BM cells were treated with no drug (ND) or cultured in the presence of IL-6 or ruxolitinib (Rux) for 14 days and expression of TGM2 was evaluated by RT-PCR. **e** Immunoblot results for TG2 from cells treated as described in (d), β-tubulin served as a loading control. **f** HME2-BM cells constructed as described in panel (a) were injected into the mammary fat pad and caliper measurements were used to quantify tumor growth. Arrow indicates initiation of neratinib or ruxolitinib or combination treatment. As a control mice were treated with an equal concentration of drug solvent (DMSO). Data are the mean ± SE of 5 mice per group resulting in the indicated *p*-values where *p < 0.05, **p < 0.01

Previous studies demonstrate that the TG2 proximal promoter contains both NF-κB and STAT3 binding elements and that TG2 expression can be driven by IL-6 [14, 22]. Consistent with these findings and our data in the previous figure, we observed decreased expression of TG2 in the HME2-BM cells when the S.R. was present (Fig. 5a). To directly interrogate the role of STAT3 in mediating TG2 expression we depleted STAT3 using an shRNA approach (Fig. 5b). Indeed, depletion of STAT3 also led to a diminution in TG2 levels (Fig. 5b). Depletion of STAT3 or co-administration of ruxolitinib also decreased the ability of HME2-BM cells to acquire neratinib resistance (Fig. 5c). Similar to depletion of STAT3, treatment of the HME2-BM cells with ruxolitinib also reduced expression of TG2 mRNA and protein (Fig. 5d-e). To evaluate the therapeutic potential of these findings we engrafted either control or STAT3 depleted HME2-BM cells onto the mammary fat pad and treated mice with neratinib, ruxolitinib or a combination of both compounds (Fig. 5f). Consistent with findings in Fig. 2, the HME2-BM cells quickly acquire resistance to neratinib, and tumors continued to grow in the presence of drug treatment (Fig. 5f). Similarly, treatment with ruxolitnib or depletion of STAT3 had no effect on tumor growth (Fig. 5f). In contrast, depletion of STAT3 or combination with ruxolitinib led to tumor regression upon treatment with neratinib (Fig. 5f). Taken together, these data clearly indicate that targeting JAK:STAT signaling in combination with ErbB inhibition can improve response to HER2-targeted therapies.

### Expression of TG2 and IL-6 predict disease recurrence in HER2 + breast cancer

**Fig. 6:**
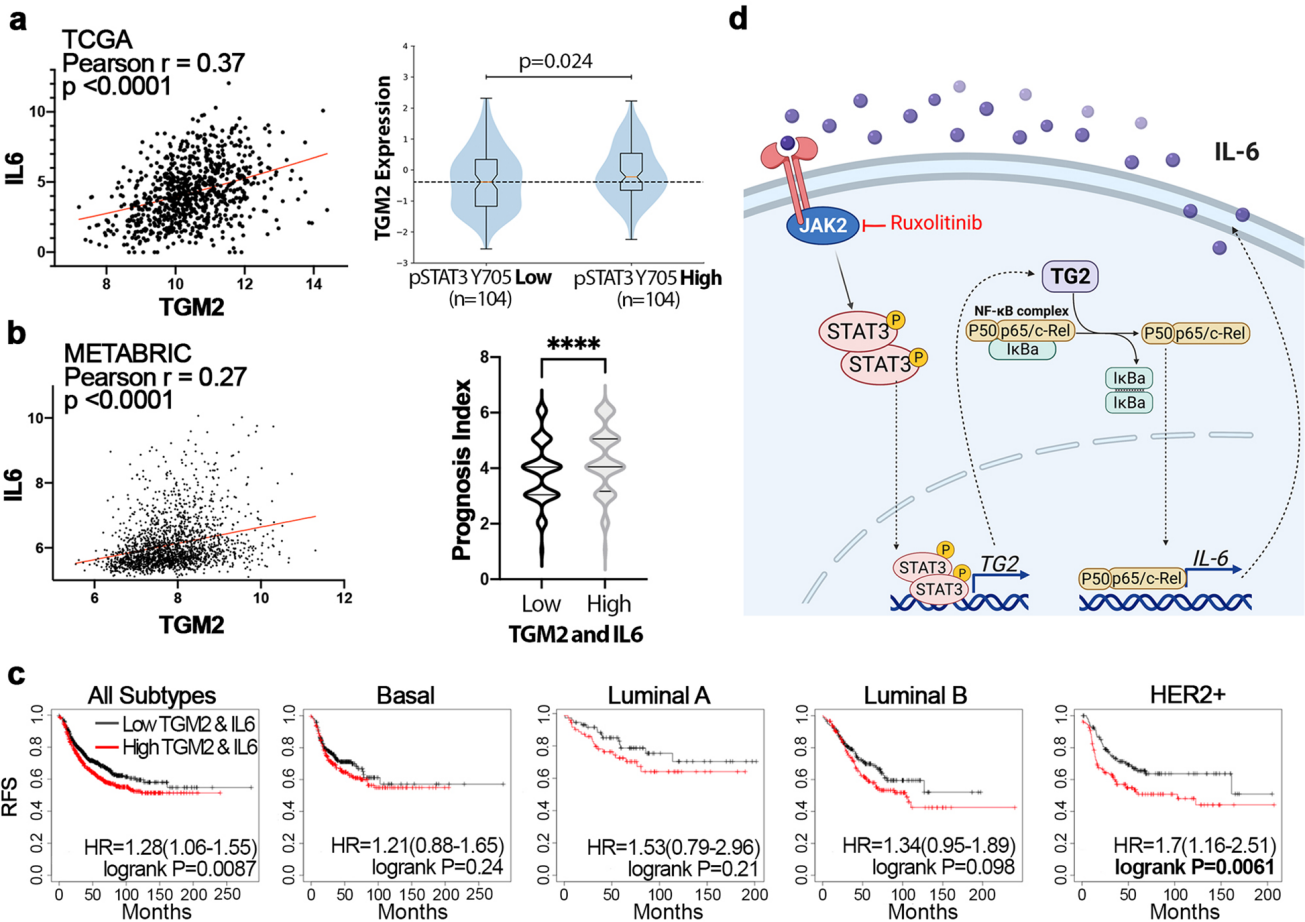
Expression of TG2 and IL6 predict disease recurrence in HER2 + breast cancer. **a** Left, Correlation analysis of the TCGA dataset comparing IL6 and TGM2. Data were analyzed using a Pearson correlation test, yielding the indicated r and *p* values. Right, comparison of TGM2 expression in patient samples bearing high (above the mean) or low (below the mean) phosphorylation of STAT3. **b** Left, Correlation analysis of the METABRIC dataset comparing IL6 and TGM2. Data were analyzed as in panel (a). Right, comparison of Nottingham Prognosis Indexes for patients with high (above the mean) or low (below the mean) expression of TGM2 and IL6. Data were analyzed by a student’s T-test where **** is *p* < 0.0001. **c** Kaplan Meier analyses for relapse free survival (RFS) of patients with high (above the mean) or low (below the mean) expression of fibronectin, TGM2, and IL6. Data were analyzed by a logrank test. **d** A schematic representation of a TG2-mediated drug persistence loop where breast cancer cells upregulate TG2 driving NF-κB-mediated expression of IL-6. Autocrine and paracrine IL-6-mediated activation of JAK:STAT3 signaling contributes to maintained expression of TG2 completing the drug persistence signaling loop. Application of the JAK2 inhibitor ruxolitinib can disrupt this persistence

To examine the clinical applicability of our findings we analyzed several patient data sets. Consistent with our findings there is a significant correlation between IL-6 and TGM2 mRNA expression across the 1084 patient samples in the TCGA invasive breast cancer dataset (Fig. 6a). Analysis of the RPPA data included in this cohort also demonstrated that TGM2 mRNA is significantly increased in patient samples that have higher levels of STAT3 phosphorylation (Fig. 6a). Similarly, analysis of the 2509 patient samples in the METABRIC dataset also demonstrated a significant correlation between IL6 and TGM2 expression (Fig. 6b). Importantly, those patients with increased levels of TGM2 and IL6 received a significantly poorer prognosis index (Fig. 6b). To further investigate the importance of these factors in disease progression we analyzed relapse free survival in patients bearing increased levels of fibronectin, TGM2, and IL6 (Fig. 6c). These markers clearly predict for decreased patient survival, and breakdown of these findings into breast cancer subtypes indicated that this significance is derived from the HER2^+^ patients (Fig. 6c). Overall, these clinical findings support and are consistent with our mechanistic data suggesting a signaling loop in which STAT3 contributes to the expression of TGM2, leading to NF-κB-mediated upregulation of IL-6 (Fig. 6d). Overall, this signaling loop appears to play a role in facilitating resistance to neratinib and potentially other HER2-targeted agents.

## Discussion

Induction of epithelial-mesenchymal transition (EMT) is associated with acquisition of drug resistance [23]. The processes of EMT lead to upregulation of bypass growth factor receptors and co-receptors that facilitate tumor growth in the presence of HER2 inhibitors [7, 24]. Indeed, previous studies from our lab demonstrate that prolonged treatment with the HER2-targeting drugs lapatinib and traztuzumab-DM1 results in the emergence of resistance cells that have upregulated fibroblast growth factor receptor (FGFR) and other mechanisms that serve as bypass pathways to achieve proliferative signaling [3, 25, 26]. However, as cells transition between these different growth programs we hypothesized that intermediate mechanisms are at play to allow cells to persist following initial drug challenge. To investigate this, we compared parental HER2-transformed cells (HME2) to their metastatic counter parts (HME-BM). These metastatic cells do not display increased expression of FGFR or other bypass mechanisms and transient dose response assays yielded similar sensitivity to HER2-targeting agents (Fig. 1) [3]. However, upon prolonged neratinib treatment, the HME2-BM metastatic cells are able to spontaneously acquire resistance, an event not observed in their parental HME2 counterparts.

Along these lines, neratinib treatment prevents tumor formation in the HME2 parental cells, but in vivo growth of HME2-BM tumors is not affected by the same treatment. These data suggest that in addition to stable autocrine mechanisms, metastatic cells may also be engaging their microenvironment to resist drug treatment. Accordingly, use of ruxolitinib or depletion of STAT3 led to regression of tumor growth in vivo, suggesting that IL-6 or possibility other inducers of JAK:STAT signaling are clearly at play during in vivo acquisition of drug resistance. Indeed, the broad anti-inflammatory properties of ruxolitinib have been demonstrated in several conditions and drive its indication in graft-versus host disease. This type of response is also consistent the lack of efficacy of ruxolitinib as a single agent in solid tumors [27, 28]. A recent study demonstrates that addition of ruxolitinib to trastuzumab did not improve response in heavily pretreated patients [29]. These clinical findings are consistent with the notion that the TG2:NF-κB:IL6:STAT3 loop is operant during acquisition of resistance, and targeting this pathway is incapable of re-sensitizing tumors that have already bypassed HER2 signaling via alternative growth factor pathways. Given that ruxoltinib is well tolerated by patients, the true clinical utility of this compound in the HER2 setting may be in upfront combination with HER2 targeted agents.

The relationship between Nf-κB:IL6:STAT3 is well established [30]. Our studies add the intracellular function of TG2 as a key component in allowing this signaling loop to persist in an autocrine fashion. Numerous inhibitors of TG2 have been developed, but they are largely focused on blocking the extracellular crosslinking function of the enzyme [31]. Additionally, small molecules are available for inhibition of Nf-κb signaling. However, several of these molecules also inhibit broad cellular functions such as nuclear transport or proteasome function [32]. Specific inhibitors of IκB kinase (IKK), the kinase responsible for Iκ-Bα phosphorylation and degradation, do exist but TG2-mediated activation of NF-κB has been shown to be independent of IKK activity [33]. Therefore, we chose to utilize the clinically approved compound ruxolitinib to break the TG2 drug persistence loop (Fig. 6d).

The use of immunocompromised mice is a drawback of our study. The acute use of ruxolitinib and its inhibition of JAK:STAT signaling leads to robust inhibition of cytokine signaling and blockade of inflammatory signaling utilized by cancer cells for tumor growth and progression. However, prolonged application of ruxolitinib is also associated with suppression of adaptive immunity. Whether this suppression of lymphoid function and immune surveillance will support emergence of underlying cancers remains to be determined [28].

The potency, covalent nature, and broad target spectrum of neratinib all contribute to its ability to effectively inhibit the growth of HER2-driven tumors. However, these characteristics also lead to significant toxicities associated with the compound. Tucatinib, a highly potent kinase inhibitor that has increased specificity for HER2, and trastuzumab-drug conjugates are emerging as additional standards of care for HER2 + patients [34]. The enhanced specificity of these compounds for HER2 decreases systemic toxicities [35]. However, the HER2 degradative properties of neratinib (Fig. 1a) suggest that the current mechanisms of persistence may be at play during any HER2-targeting event. Studies to determine if antibody-mediated drug delivery will be sufficient to overcome this persistence mechanism are currently underway in our laboratory.

## Materials and methods

### Reagents

Human mammary epithelial cells (HMLE) stably transduced with firefly luciferase and slected for using Blasticidin. These bioluminescent cells were used to construct the parental HME2 cell line via lentiviral transduction of HER2 under puromycin selection. The HME2-BM cell line was isolated from bone metastases following mammary fatpad engraftment of the HME2 cells as previously described [19]. Lentiviral transduction of pLV encoding shRNA’s targeting TGM2 or a scrambled shRNA as a control were used reduced expression of TG2 (VectorBuilder, Santa Clara, CA) encoding an. Human TGM2 or GFP as a control were also stably expressed following pLV transduction, as previously described [11]. STAT3-targeted shRNA’s (shSTAT3#1: CTCAGAGGATCCCGGAAATTT, shSTAT3#2: GGCGTCCAGTTCACTACTAAA) were similarly expressed from pLV (VectorBuilder, Santa Clara, CA). Hygromycin selection was used for stable selection of the constructs above. Stable expression of the Iκbα was achieved through lenti-viral transduction of p-Babe-GFP- Iκbα (super repressor mutant (S.R)) or p-Babe-GFP as control followed by selection using FACS. Where indicated cells were treated with every third day until drug resistance was observed. HME2-BM and HME2-TG2 cells treated with neratinib were cultured as the BMNR and TG2NR populations respectively.

### Animal experiments

IACUC approval from Purdue University was received for conducting all in vivo assays. HME2 parental and HME2-BM cells were engrafted into the duct of the second mammary fat pad of 6–8-week old female, NSG mice (2 × 10^6^ / 50 µl / mouse). When tumors reached 100 mm^3^, mice an oral gavage of neratinib (27 mg/kg/q.o.d). Neratinib was first solubilized in DMSO, and 0.25% Tween-80, 0.5% carboxymethyl cellulose solution was used for oral gavage at a final DMSO concentration of 10%. In separate experiments, female 8-week old NSG mice were engrafted with HME2-BM cells and treatment with neratinib (27 mg/kg/q.o.d) and/or ruxolitinib (40 mg/kg/q.o.d) was initiated when tumors were 100 mm^3^. The volume of mammary tumors were determined using digital calipers following the equation V = (length^2^)*(width)*(0.5).

### 3D hydrogel assays

Using a white-walled 96-well dish, 2000 cells were plated on 50 μl of solidified Cultrex basement membrane extract (BME) from (Trevigen, Gaithersburg, MD). Cells were plated in 10% FBS and 5% BME in DMEM. These cultures were treated with DMSO as a control or neratinib (100 nM) every three days. Growth was tracked every three days using bioluminescence and images were taken at Day 27.

### Immunological assays

A modified RIPA lysis buffer containing 150 mM NaCl, 50 mM Tris, 1.0% NP40, 0.1% SDS, 40 mM β-glycerolphosphate, 0.25% Sodium Deoxycholate, 10 mM activated sodium ortho-vanadate, protease inhibitor cocktail, and 20 mM sodium fluoride was used to lyse cells. Reducing, 10% SDS PAGE was used to separate proteins and membranes were probed for TG2 (Invitrogen CUB 7402, Mouse/Rat TGM2 antibody R & D systems, AF5418), phospho AKT (Ser 473) (CST Rabbit mAB#4060), total AKT (CST Rabbit 9272) phospho-STAT3 (Tyr705) (D3A7) (CST Rabbit mAB# 9145) and total STAT3 ((CST Rabbit mAB#2640), Iκbα (CST #) or β-tubulin (DSHB, Iowa City, IA). For the kinase profiling array whole cell lysates were generated from HME2-parental and HME2-BM cells under full growth media conditions. The phospho-kinase array was processed according to the manufactures instructions (R&D systems #ARY003C).

### RT-PCR analyses

Isolated total RNA (Omega bio-tek, Norcross, GA), was reverse-transcribed (Thermo Fisher) and iQ SYBR Green was used for semi quantitative real-time PCR (Thermo Fisher). The reference gene was GAPDH. The following primers were used for the analysis. Human TG2: forward: ATAAGTTAGCGCCGCTCTCC, reverse: CTCTAAGACCAGCTCCTCGG and human IL-6: forward CCAGTACCCCCAGGAGAAGA Reverse: TGTTTTCTGCCAGTGCCTCT.

### AlphaLISA IL-6 assay

IL-6 expression by each cell line was determined by using IL-6 (human) AlphaLISA detection kit. Cells were plated into 24 well plates (1 × 10^5^ cells/well). Following overnight incubation, cells were incubated with serum free DMEM media. After 24 h incubation, culture supernatants were collected, centrifuged to pellet any detached cells and IL-6 was quantified using AlphaLISA detection kit.

### Patient data analyses

Patient data from the Molecular Taxonomy of Breast Cancer International Consortium (METABRIC) and cancer genome atlas (TCGA) were accessed through cBioportal. Relapse free survival based on differential gene expression was done using KM Plotter [36].

### NF-κB Reporter assays

Cell expressing or depleted for expression of TG2 were transfected with pNifty2-Luc (InvivoGen) and pGL3 encoding renilla luciferase, containing no promoter sequence using TransIT LT1 transfection reagent (Mirus). 36 h after transfection cells were processed for renilla and firefly activity using the Dual luciferase Assay system (Promega).

### Statistical analyses

2-sided T-tests and 2-way ANOVA were used to compare two groups when the variance was similar and all other data met the assumptions of these tests. Data were considered significant when *P* values were less then 0.05. Exclusion criteria were not utilized.

## Supplementary Information

**Additional file 1: Figure S1.** Depletion of the TG2 decreases NF-kB activity.

## Data Availability

All data generated in this study are available within the manuscript and supplemental files.

## Abbreviations

MT: Empty
EMT: Epithelial-mesenchymal transition
SDS: Sodium dodecyl sulfate
NaCL: Sodium Choloride
q.o.d.: Oral gavage every other day
FACS: Fluorescence activated cell sorting
HMLE: Human mammary epithelial cells
HME2: HMLE cell transformed by HER2 overexpression
HME2-BM: HME2 cells isolated from bone metastases
IKK: IκB kinase
FGFR: Fibroblast growth factor receptor
TCGA: The Cancer Genome Atlas
METABRIC: Molecular Taxonomy of Breast Cancer International Consortium
TG2: Transglutaminase-2(Protein)
TGM2: Transglutaminase-2 (Gene)
JAK: Janus Kinase
STAT: Signal transducer and activator of transcription
HER2: Human epidermal growth factor receptor-2
NF-κB: Nuclear factor kappa B
TME: Tumor microenvironment
IL-6: Interluekin-6
S.R.: Super repressor
