# Supplementary figures and images for: Transglutaminase-2 mediates acquisition of neratinib resistance in metastatic breast cancer
Source: Mol Biomed. 2022 Jun 22;3:19. doi: 10.1186/s43556-022-00079-y (PMC9213622; doi:10.1186/s43556-022-00079-y)

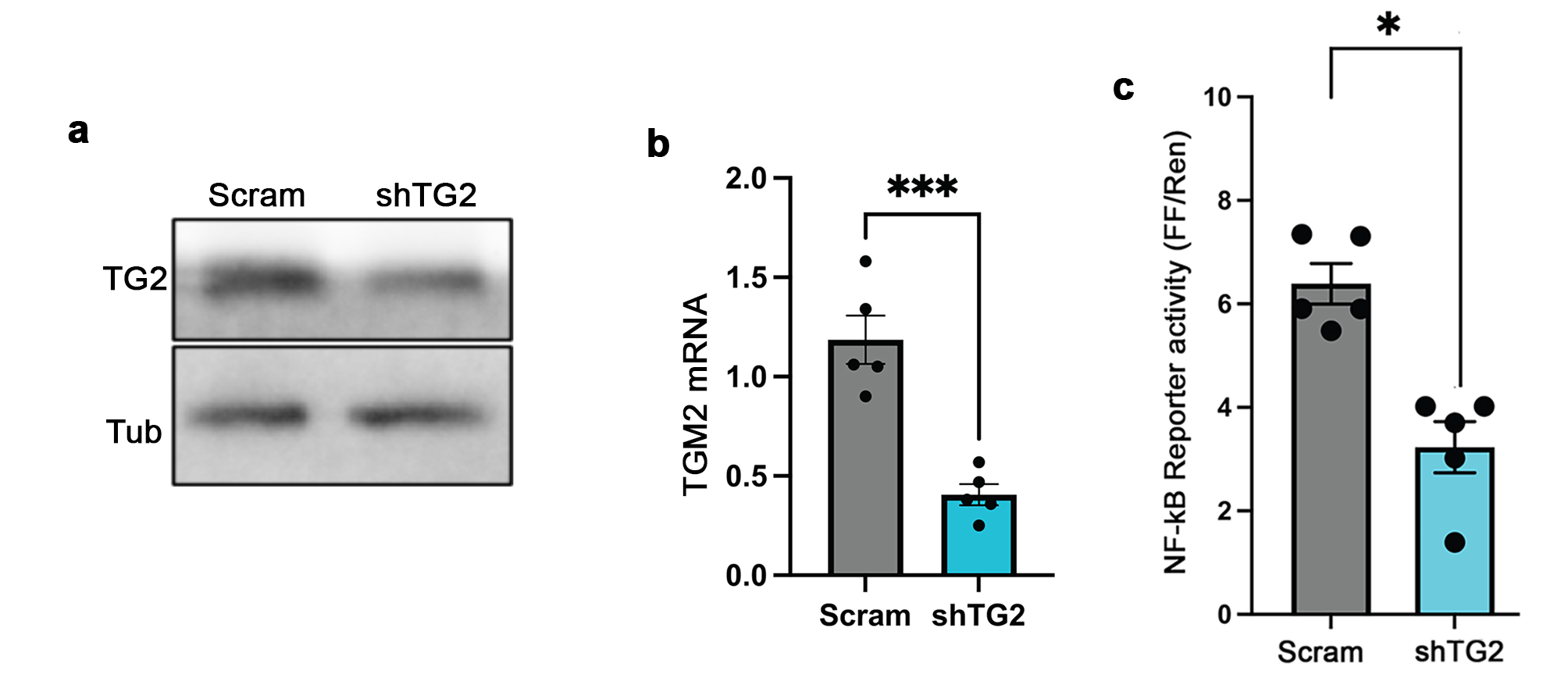

Supplement: Supplementary file 1 — Additional file 1: Figure S1. Depletion of the TG2 decreases NF-kB activity. [file 43556_2022_79_MOESM1_ESM.jpg]
